# The role of thyroid function in borderline personality disorder and schizophrenia: a Mendelian Randomisation study

**DOI:** 10.1186/s40479-024-00246-3

**Published:** 2024-02-15

**Authors:** Oladapo Babajide, Alisa D. Kjaergaard, Weichen Deng, Aleksander Kuś, Rosalie B. T. M. Sterenborg, Bjørn Olav Åsvold, Stephen Burgess, Alexander Teumer, Marco Medici, German Borderline Genomics Consortium, Christina Ellervik, Bass Nick, Panos Deloukas, Eirini Marouli

**Affiliations:** 1 Queen Mary University of London, William Harvey Research Institute, Barts and The London School of Medicine and Dentistry, London, UK; 2 Aarhus University Hospital, Steno Diabetes Center, Hedeager Aarhus, Denmark; 3 Department of Internal Medicine and Endocrinology, Medical University of Warsaw, Warsaw, Poland; 4 Erasmus Medical Center, Academic Center for Thyroid Diseases, Department of Internal Medicine, Rotterdam, Netherlands; 5 Erasmus Medical Center, Department of Epidemiology, Rotterdam, Netherlands; 6 Department of Internal Medicine, Radboud University Medical Center, Nijmegen, Netherlands; 7 Department of Public Health and Nursing, Department of Endocrinology, Clinic of Medicine, NTNU, Norwegian University of Science and Technology &, St. Olavs Hospital, Trondheim University Hospital, Trondheim, Norway; 8 University of Cambridge, MRC Biostatistics Unit, Cambridge Institute of Public Health, Cambridge, UK; 9 Cardiovascular Epidemiology Unit, Department of Public Health and Primary Care, University of Cambridge, Cambridge, UK; 10 Institute of Community Medicine, University Medicine Greifswald, Greifswald, Germany; 11 DZHK German Center for Cardiovascular Research, Berlin, Germany; 12 Department of Laboratory Medicine, Boston Children’s Hospital, Boston, MA USA; 13 Department of Pathology, Harvard Medical School, Boston, MA USA; 14 Faculty of Health and Medical Sciences, Department of Clinical Medicine, University of Copenhagen, Copenhagen, Denmark; 15 Division of Psychiatry, University College London, Mental Health Neuroscience, London, UK

**Keywords:** Schizophrenia, Borderline Personality Disorder, Mendelian Randomisation, Thyroid function, TSH, FT4

## Abstract

**Background:**

Genome-wide association studies have reported a genetic overlap between borderline personality disorder (BPD) and schizophrenia (SCZ). Epidemiologically, the direction and causality of the association between thyroid function and risk of BPD and SCZ are unclear. We aim to test whether genetically predicted variations in TSH and FT4 levels or hypothyroidism are associated with the risk of BPD and SCZ.

**Methods:**

We employed Mendelian Randomisation (MR) analyses using genetic instruments associated with TSH and FT4 levels as well as hypothyroidism to examine the effects of genetically predicted thyroid function on BPD and SCZ risk. Bidirectional MR analyses were employed to investigate a potential reverse causal association.

**Results:**

Genetically predicted higher FT4 was not associated with the risk of BPD (OR: 1.18; *P* = 0.60, IVW) or the risk of SCZ (OR: 0.93; *P* = 0.19, IVW). Genetically predicted higher TSH was not associated with the risk of BPD (OR: 1.11; *P* = 0.51, IVW) or SCZ (OR: 0.98, *P* = 0.55, IVW). Genetically predicted hypothyroidism was not associated with BPD or SCZ. We found no evidence for a reverse causal effect between BPD or SCZ on thyroid function.

**Conclusions:**

We report evidence for a null association between genetically predicted FT4, TSH or hypothyroidism with BPD or SCZ risk. There was no evidence for reverse causality.

**Supplementary Information:**

The online version contains supplementary material available at 10.1186/s40479-024-00246-3.

## Background

The thyroid gland controls metabolic functions and upholds an intricate equilibrium of energy in the organism. Thyroid function is routinely assessed by plasma (or serum) levels of thyroid-stimulating hormone (TSH) and free thyroxine (FT4). Abnormal activity within the thyroid has been linked to several psychological conditions, such as depression, anxiety, and bipolar disorder [1]. We have previously shown evidence of a causal association of thyroid function with risk of Alzheimer's disease [2] but not with depression [3]. Cross-disorder genomic analyses have shown that psychiatric disorders such as SCZ are inter-linked to bipolar disorder, obsessive–compulsive disorder and autism spectrum disorders [4]. The latest genome-wide association study (GWAS) for borderline personality disorder (BPD) revealed a genetic overlap with bipolar disorder, major depression and SCZ [5, 6].

BPD is a personality disorder defined by negative patterns of insecurity in emotion management, interpersonal interactions, identity, and impulse control [7, 8]. BPD is one of the most common personality disorders, and it has been observed that 15–20% of the psychiatric outpatient population is being diagnosed with BPD [7, 8]. BPD has also been linked with an increased prevalence of suicidal behaviours. The heredity of BPD has been estimated using twin studies, with recent estimates of up to 46% [9]. Auditory hallucinations, cognitive abnormalities, delusions, and other symptoms, such as social withdrawal, are common in SCZ [10]. Patients typically develop symptoms of SCZ in their early adult years. It is evident in twin studies that the lifetime risk of SCZ for monozygotic twins is above 40%, substantially higher than the 1% lifetime risk seen in the general population [10, 11]. Several studies have highlighted connections between SCZ and various other psychiatric disorders, including hyperactivity disorder, anorexia nervosa. anxiety disorder, autism spectrum disorder, bipolar disorder, depressive disorder, obsessive–compulsive disorder and posttraumatic stress disorder [12–14].

There have been several studies investigating the links between thyroid function and mental disorders. Additional studies from hospitalised patients revealed that, in addition to bipolar disorder, SCZ is linked to hypothyroidism, with hypothyroidism being reported in 25.17% of patients with schizophrenia-spectrum disorders [15, 16]. Thyroid hormones and their relationships with neurotransmitter systems appear to have a significant role in SCZ too [17]. Notably, it has been reported that hypothyroid patients seem to have reduced serotonin responsiveness, which is reversible in thyroid hormone replacement therapy [18]. A population-based study demonstrated an independent association between hypothyroidism and SCZ [19], along with other studies which have found that FT4 levels were lower in patients with SCZ [20].Therefore, in the current study, we aimed to explore the bi-directional causal associations of thyroid function with BPD and SCZ using a Mendelian Randomization design.

## Materials and methods

### Availability of data and materials

#### Summary statistics and study populations

The datasets on thyroid function were derived from the ThyroidOmics Consortium [21, 22]. Additional analyses for TSH were performed using estimates from GWAS data for the full range TSH levels (meta-analysis of ThyroidOmics, HUNT and MGI studies) in individuals younger than 50 years old, and TSH within the normal and full range (from the HUNT study). Further analyses for hypothyroidism included 30,234 individuals with autoimmune thyroid disorder (mostly hypothyroidism) and 725,172 controls [23]. We used data from GWAS studies, more specifically: 88 genetic variants for TSH within the normal range [22, 24], 31 for FT4 [24] and 96 for hypothyroidism [23]. (Additional file 1: sheet: Stables 1a-1 g, Additional file 2: sheet: Stables 1a-1 g). The summary statistics for borderline personality disorder disease (1075 BPD patients and 1675 controls) were obtained from the German Borderline Genomics Consortium [6]. The summary statistics for SCZ disease were obtained from the Working Group of the Psychiatric Genomics Consortium involving a meta-analysis including up to 76,755 individuals with schizophrenia and 243,649 control individuals [25].

### Two-sample Mendelian Randomisation

Two-sample MR (*2SMR*) analyses were performed using data from large GWAS summary statistics. A two-sample MR package is available in R [26], and we implemented in R 4.0.2 version. We employed the Inverse Variance Weighted (*IVW*) method, where the IVW estimator assumes that there is no pleiotropy, and it maximises the likelihood function and achieves minimal variance [27]. Additional analyses were conducted by utilising MR-Egger [28], weighted median (*WM*) [29] and MR Pleiotropy RESidual Sum and Outlier (*MR-PRESSO*) [30]. *WM* relaxes the first IV assumption, as it assumes that at least 50% of the weight contributed by genetic variants comes from valid instrumental variables (IVs). The *MR-Egger* intercept is used to test for directional pleiotropy and can be interpreted as the average pleiotropic effect across all IVs. One of the main assumptions of 2SMR is that no horizontal pleiotropy should be present [31]. When this assumption is violated, the analysis and results can become heavily distorted, leading to bias and potentially outputting false-positive causal relationships. *MR-PRESSO* utilises three components to detect the presence/absence of horizontal pleiotropy: (a) detection of horizontal pleiotropy (global test), (b) removal of outliers to correct for horizontal pleiotropy (outlier test); and (c) testing of significant differences in the causal estimates before and after correction for outliers (distortion test). We adopted the MR Steiger directionality test to test the causal directionality test [32]. The established association is considered directionally reliable if the IVs explain more of the variation (r^2^) in the outcome than the exposure [33]. A detailed description of this methodology has been described previously [34].

We applied Mendelian Randomisation (MR) approaches using genetic instruments associated with TSH and FT4 levels as well as hypothyroidism to examine the effects of genetically predicted thyroid function on BPD and SCZ risk. Additional analyses examined whether genetic predisposition to hypothyroidism is causally associated with the risk of developing SCZ or BPD disease, along with evaluation of the role of thyroid function with estimates derived from TSH GWAS in individuals with TSH within the normal or full range as well as individuals younger than 50 years old. We further performed sensitivity analyses using FT4-associated genetic variants on *DIO1* and *DIO2* genes along with leave-one-out analyses. Bidirectional MR analyses were performed to investigate a potential reverse causal association, i.e., whether genetic predisposition to BPD and/or SCZ may have a causal effect on thyroid function.

## Results

**Fig. 1:**
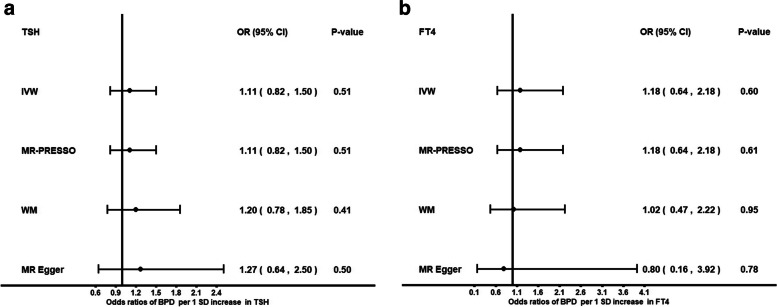
Forest plot: Odds ratios for the effect of genetically predicted TSH **a** FT4 (excluding rs4842131, rs2235544) **b** on BPD risk. CI, confidence interval; fT4, free thyroxine; IVW, inverse variance weighted; MR, Mendelian randomization; MR-PRESSO, MR Pleiotropy RESidual Sum and Outlier; OR, odds ratio; SD, standard deviation; WM, weighted median; TSH, Thyroid stimulating hormone; BPD: Borderline Personality Disorder

**Fig. 2:**
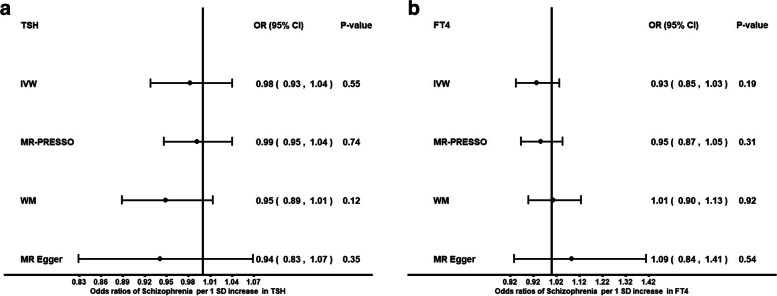
Forest plot: Odds ratios for the effect of genetically predicted TSH **a** FT4 (excluding rs4842131, rs2235544) **b** on SCZ risk, CI, confidence interval; fT4, free thyroxine; IVW, inverse variance weighted; MR, Mendelian randomisation; MR-PRESSO, MR Pleiotropy RESidual Sum and Outlier; OR, odds ratio; SD, standard deviation; TSH, Thyroid stimulating hormone; WM, weighted median; SCZ, Schizophrenia

**Fig. 3:**
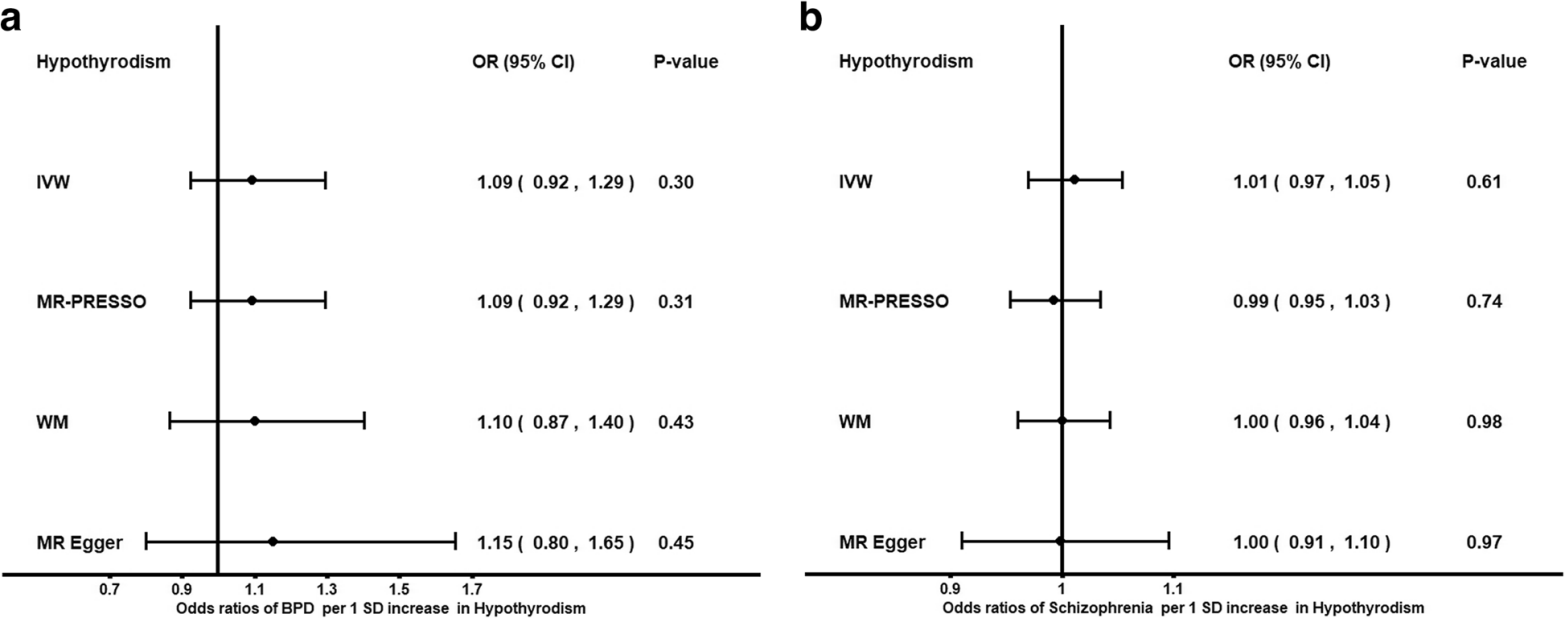
Forest plot: Odds ratios for the effect of genetically predicted hypothyroidism on BPD **a** and SCZ **b** risk. CI, confidence interval; IVW, inverse variance weighted; MR, Mendelian randomization; MR-PRESSO, MR Pleiotropy RESidual Sum and Outlier; OR, odds ratio; SD, standard deviation; WM, weighted median, BPD: Borderline Personality Disorder, SCZ: Schizophrenia

Genetically predicted higher FT4 levels were nominally associated with decreased risk of BPD (OR: 0.49; *P* = 0.03, weighted median [WM] MR). This finding was only observed with the WM method. As it is unusual for the WM method to have such a high discrepancy with the IVW method, we further explored this association by performing leave-one-out analyses. These analyses highlighted that the signal was mainly driven by two genetic variants, rs4842131 in *LHX3* and rs2235544 in *DIO1* (See Additional file 1: (sheet: STable7)). After removing these two variants, the results were concordant across methods towards null association. Genetically predicted higher TSH was not associated with the risk of BPD (OR: 1.2; *P* = 0.41, WM). *MR-PRESSO* did not identify potential outliers. There was no evidence for an association between genetically predicted hypothyroidism and BPD (OR: 1.1, *P* = 0.43) (See Additional file 1: (sheet: STables 2–3, 8), Figs. 1a-b, 3b). We also observed a nominally significant association between genetically predicted FT4 and SCZ with the WM method. We further explored the observed nominal association between FT4 and SCZ with the WM method by performing leave-one-out analyses, as there was some heterogeneity in the findings across methods. Leave-one-out analyses indicated that the same two variants, rs4842131 (LHX3*)* and rs2235544 (*DIO1*), were driving the association (See Additional file 2: sheet: STable6). After excluding those variants, our results suggest no association between increased FT4 levels and the risk of SCZ (OR: 0.93; *P* = 0.19, IVW). *MR- PRESSO* analysis on corrected outliers did not show casual association (OR: 0.95; *P* = 0.3).In addition, 2SMR analyses were conducted to investigate the presence of an association between TSH levels, hypothyroidism and SCZ, and there was no evidence for causal association (OR: 0.98; *P* = 0.55) and (OR: 1.00, *P* = 0.61), respectively (See Additional file 2: (sheet: STables 2–3,6–7), Figs. 2a-b, 3a).

Additional analyses using data from GWAS for full range TSH levels (HUNT study), TSH levels (HUNT study with participants younger than 50 years old), TSH levels (HUNT with TSH within normal range), TSH levels (meta-analysis of HUNT, MGI, ThyroidOmics data) revealed no association between thyroid function and BPD or SCZ (See Additional file 1 and 2: (Sheet: STable4)).

A causal direction test (MR Steiger) was performed on all associations to determine if there was any reverse causality. For the most significant initial signal between FT4 and BPD (WM), the additional tests suggest the direction of the already identified causal direction (*P* = 0.0002) (See Additional file 1: (sheet: STable2)). The direction of the nominally significant effect FT4 and SCZ were also confirmed (*P* = 9.15e-261) (See Additional file 2: (sheet: STable2)). Bidirectional MR was carried out to address a potential reverse causal association and investigate whether SCZ or BPD have a causal effect on TSH or FT4 levels. We performed a reverse MR using data from the latest BPD and SCZ GWAS and selecting variants after pruning at r^2^ = 0.001. There was no evidence of causality between genetically predicted BPD or SCZ with thyroid function (See Additional file 1: (sheet: STable5)), See Additional file 2: (sheet: STable5)). We further evaluated whether any association between FT4 and BPD is driven by only the deiodinase-associated variants, but the signal was null (OR = 0.55, *P* = 0.2) (See Additional file 1: (sheet: Stable6)).

## Discussion

In this study, extensive MR analyses were conducted to assess the potential causal role of thyroid function on BPD and SCZ risk. MR results suggest that genetically predicted thyroid function evaluated through FT4, TSH or hypothyroidism is not associated with the risk of BPD or SCZ. Severe childhood trauma-related stress could promote lasting altered thyroid levels through effects on the hypothalamic pituitary thyroid axis and contribute to the development of psychopathology associated with BPD traits [35]. Even though there is epidemiological evidence suggesting improvement in BPD symptoms after administering higher than normal levels of FT4 in women [18], our results did not detect direct evidence for a causal association. We initially detected a signal between FT4 and BPD or SCZ that was largely attributed to two genetic variants in the *LHX3* and *DIO1* genes, respectively. *LHX3* is a transcription factor that modulates the expression of multiple genes implicated in the pituitary gland and nervous system development [36]. Variants in the *LHX3* gene may alter the responsiveness of the pituitary gland to TSH, which may subsequently affect the production of thyroid hormones.

Mutations in this gene cause combined pituitary hormone deficiency 3. In addition, phenome-wide association studies report associations with sex hormones—particularly SHBG and testosterone, body impedance measures, anthropometry, haematological parameters and other [37]. There is also evidence that SCZ might be related to abnormalities of the HPA axis. *LHX3* and early growth response 1 (*EGR1*) genes can affect pituitary function, and synapsin 2 (*SYN2*) gene can regulate the activity of *LHX3* and EGR1, which might be involved in the pathway of SCZ development [38].

The protein encoded by the *DIO1* gene belongs to the iodothyronine deiodinase family. This protein provides most of the circulating T3, which is essential for growth, differentiation, and basal metabolism in humans. There is evidence that a *DIO1* variant, rs11206244, which is in tight linkage disequilibrium with rs2235544, is associated with lifetime major depression. These findings suggest that genetic variation in HPT axis genes might be involved in the aetiology of major depression [39].

Taken together, these variants demonstrate pleiotropic effects that might be affecting BPD or SCZ through complex mechanisms rather than directly through FT4. The exclusion of these variants resulted in a null signal for both BPD and SCZ. There are several strengths in using MR as a method to find causal relations and reduce confounding. The summary statistics used were from large GWAS published to increase statistical power. MR approaches are becoming more prevalent in investigating non-confounded causal associations due to the many advantages over traditional observational epidemiological studies. The largest GWAS on BPD available has a limited sample size; thus, our ability to reverse associations between FT4 and BPD could be limited. Future GWAS studies with more specified phenotypes would increase the power to further explore causal associations and the role of potential mediators through multivariate MR analyses. It is important to note that through MR analyses, we evaluated the effects of genetically predicted thyroid function on the disease, and all phenotypes and disorders explored here are complex and not only determined by genetics. Another potential limitation of our study is that we focused our analyses on individuals of European ancestry, and studies in other ancestry populations are lacking. Future research would further clarify any potential causal role of thyroid function in individuals with BPD or SCZ. Further research is required to better understand the underlying mechanisms and the genetic correlations through which thyroid-associated variants could affect BPD or SCZ and any pathways not directly driven by thyroid function.

## Conclusion

In conclusion, our results show no evidence of bi-directional causal associations between genetically predicted FT4, TSH or hypothyroidism and BPD or SCZ.

### Supplementary Information

**Additional file 1.**

**Additional file 2.**

**Additional file 3.**

## Data Availability

All relevant data generated or analysed during this study are included in this published article and its supplementary information files.

## Abbreviations

BPD: Borderline personality disorder
FT4: Free thyroxine
GWAS: Genome-wide association study
IVW: Inverse Variance Weighted
MR: Mendelian Randomisation
MR-PRESSO: Mendelian Randomization Pleiotropy RESidual Sum and Outlier
OR: Odds Ratio
SCZ: Schizophrenia
TSH: Thyroid-stimulating hormone
2SMR: Two-sample MR
WM: Weighted median
