# Supplementary material for: The role of thyroid function in borderline personality disorder and schizophrenia: a Mendelian Randomisation study
Source: Borderline Personal Disord Emot Dysregul. 2024 Feb 15;11:2. doi: 10.1186/s40479-024-00246-3 (PMC10868101; doi:10.1186/s40479-024-00246-3)
Supplement: Supplementary file 3 — Additional file 3. [file 40479_2024_246_MOESM3_ESM.docx]

Supplementary Data 1: Flowcharts of forward (a) and reverse (b) Mendelian Randomisation Analyses


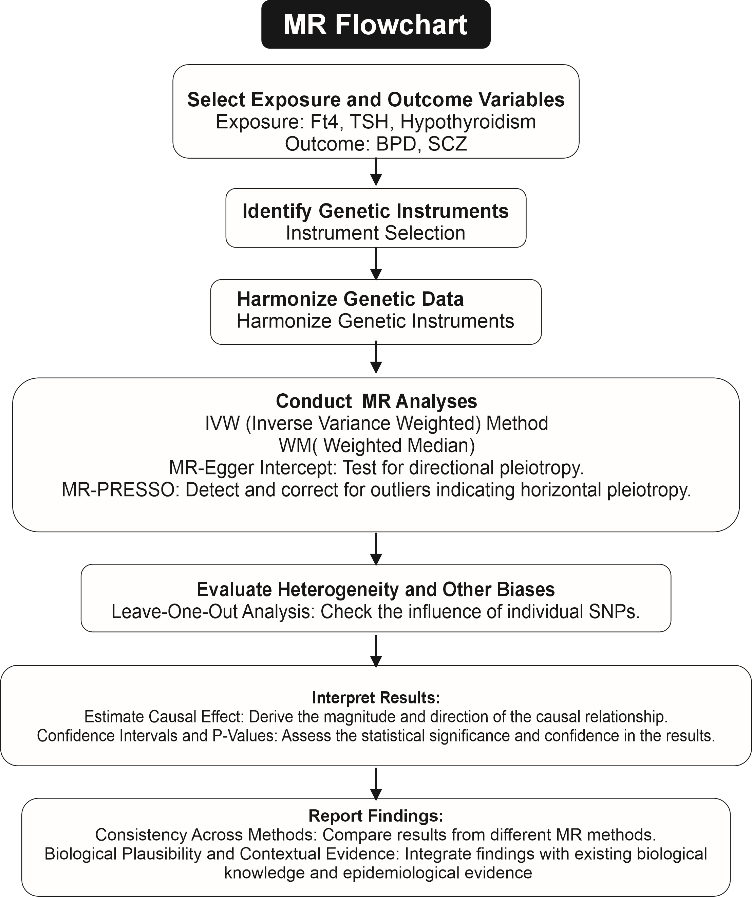


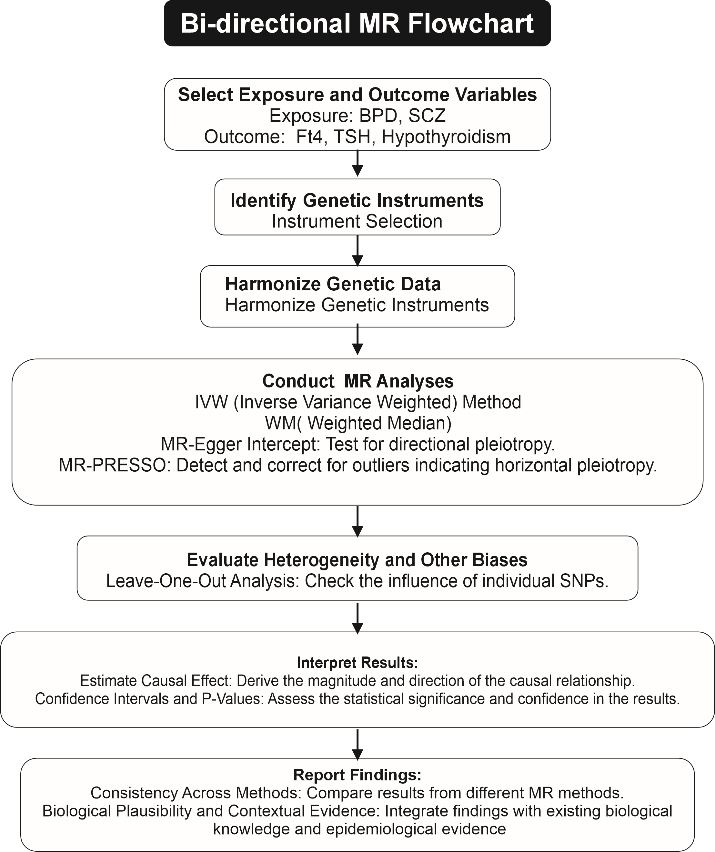


Supplementary Data 2: Details of datasets

| **Dataset Category** | **References and information** |
| --- | --- |
| TSH & FT4 levels | Teumer A, Chaker L, Groeneweg S, Li Y, Di Munno C, Barbieri C, et al. Genome-wide analyses identify a role for SLC17A4 and AADAT in thyroid hormone regulation. Nat Commun. 2018 Oct 26;9:4455.  ThyroidOmics Consortium- Teumer et al.  Sample size: up to 72,167 individuals.  Ancestry: European ancestry  Zhou W, Brumpton B, Kabil O, Gudmundsson J, Thorleifsson G, Weinstock J, et al. GWAS of thyroid stimulating hormone highlights pleiotropic effects and inverse association with thyroid cancer. Nat Commun. 2020 Aug 7;11(1):3981.  HUNT-MGI- ThyroidOmics Consortium-Zhou et al.  GWAS meta-analysis for TSH levels on the population-based Nord-Trøndelag Health Study (HUNT study) (N = 55,342), Michigan Genomics Initiative (MGI, N = 10,085), and the ThyroidOmics consortium (up to N = 54,288 samples).  Ancestry: European ancestry |
| Hypothyroidism | Saevarsdottir S, Olafsdottir TA, Ivarsdottir EV, Halldorsson GH, Gunnarsdottir K, Sigurdsson A, et al. FLT3 stop mutation increases FLT3 ligand level and risk of autoimmune thyroid disease. Nature. 2020 Aug;584(7822):619–23.  30,234 cases and 725,172 controls from Iceland and the UK Biobank |
| Borderline Personality Disorder (BPD) | Witt SH, Streit F, Jungkunz M, Frank J, Awasthi S, Reinbold CS, et al. Genome-wide association study of borderline personality disorder reveals genetic overlap with bipolar disorder, major depression and schizophrenia. Transl Psychiatry. 2017 Jun;7(6):e1155–e1155.  1075 BPD Cases,1675 controls  Ancestry: European ancestry |
| Schizophrenia (SCZ) | Trubetskoy V, Pardiñas AF, Qi T, Panagiotaropoulou G, Awasthi S, Bigdeli TB, et al. Mapping genomic loci implicates genes and synaptic biology in schizophrenia. Nature. 2022 Apr;604(7906):502–8.  Meta-analysis including 76,755 individuals with schizophrenia, 243,649 control individuals.  These are summary statistics from the meta-analysis of European ancestry cohorts. |
